# New Year’s Resolution or Spring Feelings? Effect of Month and Season on First Presentation and Short-Term Outcome in Metabolic and Bariatric Surgery

**DOI:** 10.1007/s11695-026-08665-7

**Published:** 2026-04-16

**Authors:** Lena Seidemann, Annika Hoyer, Arne Dietrich, Sjaak Pouwels, Carolina Pape-Köhler

**Affiliations:** 1 Department of Visceral, Transplant and Vascular Surgery, University Hospital Leipzig, Leipzig, Germany; 2 Biostatistics and Medical Biometry, Bielefeld University, Bielefeld, Germany; 3 Department of Surgery, Klinikum Lippe, Detmold, Germany; 4 Department of Intensive Care Medicine, Elisabeth-TweeSteden Ziekenhuis, Tilburg, Netherlands; 5 Bariatric Clinic, Klinikum Bielefeld, Bielefeld, Germany; 6 Department of General and Visceral Surgery, Bielefeld University, Bielefeld, Germany

**Keywords:** Metabolic bariatric surgery, Sex differences, Seasonality, Body image, New Year’s resolutions, StuDoQ, Patient motivation

## Abstract

**Background:**

While seasonal fluctuations in health behaviors such as dieting are well known, little is known about their influence on metabolic bariatric surgery (MBS) consultation timing. This study examines whether the season of first clinical presentation for MBS differs by sex and whether timing impacts early outcomes.

**Materials and methods:**

Data from 21,396 patients in the German StuDoQ/MBE registry (2014–2019) were analyzed. Monthly and seasonal presentation probabilities were compared by sex. Logistic regression assessed predictors of early-year consultation. Linear mixed models with random intercepts were used to evaluate the effect on weight loss at 3 and 12 months postoperatively.

**Results:**

Presentation patterns differed significantly between sexes. Women presented more frequently in spring and summer, while men showed a clear peak in the first quarter, aligning with calendar-based triggers like New Year’s resolutions. These trends suggest sex-specific motivators: body image awareness in women versus functional limitations and external cues in men. Despite seasonal variation in consultation timing, postoperative weight loss outcomes showed no statistically significant differences across all seasons.

**Conclusion:**

The timing of MBS consultation might be influenced by seasonal and sex-specific behavioral patterns. However, outcomes are unaffected by the time of year patients enter treatment. Recognizing these trends can inform tailored patient communication and engagement strategies. Future bariatric programs may benefit from sex-sensitive, seasonally adaptive approaches to optimize patient readiness and long-term success.

**Supplementary Information:**

The online version contains supplementary material available at 10.1007/s11695-026-08665-7.

## Key Points

Women present more frequently in spring/summer, likely driven by body image concerns.

Men show a peak in early-year presentations, potentially linked to New Year’s resolutions.

Seasonal timing does not significantly affect postoperative weight loss outcomes.

Personalized and seasonally aware patient counseling could be recommended.

**Supplementary Information:**

The online version contains supplementary material available at 10.1007/s11695-026-08665-7.

## Introduction

Seasonal variations and psychosocial dynamics are important modifiers of health-related behavior, including dietary change and weight management. Every year in January, public attention toward weight reduction resurfaces, coinciding with the phenomenon of New Year’s resolutions [1, 2]. This is usually reflected by online search trends, (increased) gym attendance and dietary supplement purchases [2]. However, whether this translates into an increased demand for metabolic bariatric surgery (MBS) remains unclear. Simultaneously, body image awareness—particularly among women—fluctuates seasonally. Warmer months, characterized by lighter clothing and intensified media emphasis on idealized body shapes, may heighten dissatisfaction and prompt health-seeking behavior [3–5]. Contrarily, men often show that functional limitations rather than aesthetic concerns are their main motivator. This typically requires an external trigger such as physician advice or the symbolic beginning of a new year [6, 7].

Within the German healthcare system, candidates for MBS generally undergo a structured multimodal preoperative program comprising nutritional counseling, behavioral therapy, and physical exercise, lasting 3–6 months depending on BMI and comorbidities [8]. Thus, a first consultation early in the year may result in surgery being scheduled for spring or summer. These seasonal dynamics could therefore influence both presentation timing and perceived outcomes. Despite growing interest in sex differences and behavioral determinants in MBS, few studies have examined temporal patterns in patient engagement or whether consultation timing affects postoperative outcomes [9–11]. Cambras et al. conducted a study that reported seasonal variations in the numbers and outcomes of MBS, with slightly better weight loss after summer procedures [9]. However, the influence of the time of first presentation has not been systematically evaluated.

This study aimed to [1] examine whether monthly and seasonal patterns exist in the timing of first MBS consultations [2], explore sex-specific differences in these trends, and [3] evaluate whether presentation timing is associated with early postoperative weight loss. We hypothesized that early-year presentation (January–March) would be more common, particularly among men, and that earlier presentation might be associated with slightly better weight loss outcomes.

## Materials and Methods

This study was reported in accordance with the Strengthening the Reporting of Observational Studies in Epidemiology (STROBE) guidelines [12] and adheres to the principles laid down in the 1964 Declaration of Helsinki.

### Selection of the Study Cohort

StuDoQ/MBE (Studien-, Dokumentations- und Qualitätszentrum der Deutsche Gesellschaft für Allgemein und Viszeralchirurgie/Metabolische und Bariatrische Erkrankungen) is a prospective registry on German MBS patients. Data entry is mandatory for certified German MBS centers. Patients’ informed consent is obtained before data entry. After a formal application, data were exported in December 2024. The registry excerpt included data on patients with a minimum age of 18 who underwent sleeve gastrectomy (SG) or Roux-en-Y gastric bypass (RYGB) as a primary procedure, without any reported major intra- or postoperative complications. Further inclusion criteria were available dates of first presentation at an MBS center and of the operation. After quality control, data sets containing implausible observations (BMI at baseline < 30 or > 85 kg/m^2^; BMI at follow-up < 18 or > 85 kg/m^2^) or missing values for baseline or outcome variables were excluded. Moreover, patients who first presented or were operated during the Christmas holiday season (Dec 24 to Jan 6) were excluded because only few German MBS centers maintain their routine processes during this period.

### Definition of Outcome Measurements and Study Selection Procedures

The primary outcome of interest was the probability of first presentation in the first quarter of the year (January–March). The secondary outcome was postoperative weight loss over time, measured as absolute weight loss (kg) at 3 and 12 months after surgery. In addition, descriptive analyses were performed to examine the distribution of first presentation and surgery across calendar months and seasons. First presentation was defined as the first visit to the bariatric surgical clinic. To reduce heterogeneity in postoperative recovery and follow-up patterns, we excluded patients with major complications, which could otherwise confound the assessment of seasonal effects on presentation timing and early weight-loss outcomes. The seasons were defined as follows.

- **Spring**: March, April, May (starts March 1 st, ends May 31 st).
- **Summer**: June, July, August (starts June 1 st, ends August 31 st).
- **Autumn (Fall)**: September, October, November (starts September 1 st, ends November 30th).
- **Winter**: December, January, February (starts December 1 st, ends February 28th/29th).

### Statistical Analysis

Patient characteristics were first analyzed descriptively, reporting mean and standard deviation for continuous variables and proportions for categorical variables. For quantifying associations between patient characteristics and the probability of first presentation in the first quarter of the year, logistic regression was applied. The model was adjusted for age and BMI at baseline (measured on a continuous scale), as well as sex (female vs. male), presence of depression (yes/no), and presence of a primary indication for surgery (yes/no), which were included as categorical variables. Male sex, absence of depression, and absence of a primary indication were used as reference categories. Adjustment for clustering at the center level was not possible because center identifiers were not available in the registry extract used for this analysis. For analysis of weight loss in the full cohort data with follow-up visits, a linear mixed regression model with absolute weight loss compared to baseline (in kg) as outcome was used that accounts for the relevant exposures (time of first presentation and time of operation) as well as potential time-fixed (sex, operation technique) and -varying confounding variables (age, BMI, depression, presence of type 1 or type 2 diabetes and joint pain, all measured at baseline, 3 months and 12 months postoperatively). Depression, type 1 or type 2 diabetes, and joint pain were included as time-varying covariates to account for changes in patients’ clinical status that may influence postoperative weight loss independently of seasonal presentation patterns. To account for repeated postoperative measurements within individuals (3 and 12 months postoperatively) and potential intra-individual correlations, a random intercept at patient-level was included. Results are given as effect estimates (regression coefficients, odds ratios (OR)) with 95% confidence interval and p-value where appropriate. A p-valu.

e ≤ 0.05 was considered statistically significant. A complete case analysis has been performed. The statistical software SAS 9.4 (SAS Institute Inc., Cary, NC) was used for data preprocessing the statistical analysis.

## Results

**Fig. 1:**
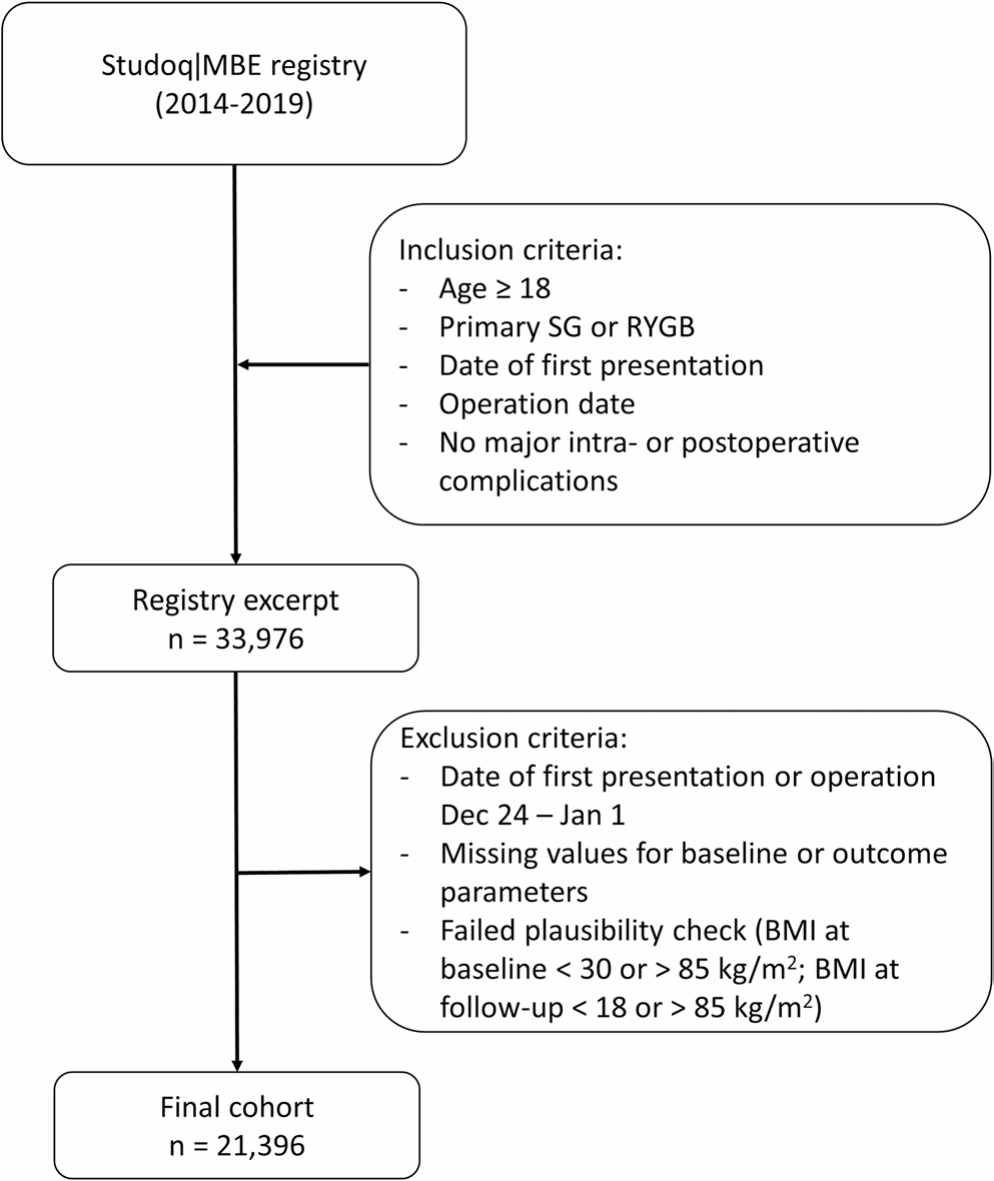
Flow chart of study cohort selection. Abbreviations: SG = Sleeve Gastrectomy; RYGB = Roux-en-Y Gastric Bypass; BMI = body mass index

The registry excerpt provided by Studoq/MBE included data from 33,976 patients. After excluding participants with implausible/missing values (*n* = 5040) and those from patients first presenting (*n* = 3850) or being operated during the Christmas season (*n* = 3150), the final number of patients included in this study was 21,396 (Fig. 1).

### Baseline Characteristics

The mean age was 43 years (SD: 11.46), 74.96% were females. Mean baseline BMI was 49 kg/m^2^ (SD: 7.54). The performed procedures were SG in 60.45%, proximal RYGB in 39.08% and distal RYGB in 0.47% of cases (Table 1).

**Table 1:** Baseline characteristics and postoperative outcomes of the study cohort

Variable	Female (*n* = 16039)	Male (*n* = 5357)	Overall (*n* = 21396)
Age at surgery, mean (SD), years	42.63 (11.35)	45.16 (11.60)	43.26 (11.46)
Proximal RYGB, No. (%)	6715 (41.87%)	1647 (30.74%)	8362 (39.08%)
Distal RYGB, No. (%)	82 (0.51%)	19 (0.35%)	101 (0.47%)
SG, No. (%)	9242 (57.62%)	3691 (68.90%)	12,933 (60.45%)
First presentation in Jan-Mar, No (%)	4111 (25.63)	1468 (27.40%)	5579 (25.63%)
Delay between first presentation and surgery, mean (SD), months	9.18 (6.93)	9.54 (7.67)	9.26 (7.12)
BMI, mean (SD), kg/m^2^			
Baseline	48.58 (11.35)	50.27 (7.91)	49.01 (7.54)
3 months	39.22 (6.66)	40.20 (7.12)	39.47 (6.79)
12 months	32.56 (6.31)	34.41 (6.63)	33.02 (6.44)
Body weight, mean (SD), kg			
Baseline	134.05 (22.43)	162.64 (28.19)	141.21 (27.01)
3 months	108.24 (19.93)	130.05 (24.89)	113.70 (23.28)
12 months	89.84 (18.41)	111.27 (28.19)	95.20 (21.66)
Depression, No. (%)			
Baseline	4964 (30.95%)	1153 (21.52%)	6117 (28.59%)
3 months	3787 (21.61%)	856 (15.98%)	4643 (21.70%)
12 months	3572 (22.27%)	849 (15.85%)	4421 (20.66%)
Type 1 Diabetes, No. (%)			
Baseline	108 (0.67%)	46 (0.86%)	154 (0.72%)
3 months	108 (0.67%)	55 (1.03%)	163 (0.76%)
12 months	102 (0.64%)	48 (0.90%)	150 (0.70%)
Type 2 Diabetes, No. (%)			
Baseline	3528 (22.00%)	1614 (30.13%)	5142 (24.03%)
3 months	3002 (18.72%)	1399 (26.12%)	4401 (20.57%)
12 months	2389 (14.89%)	1160 (21.65%)	3549 (16.59%)
Joint pain, No. (%)			
Baseline	12,209 (76.12%)	3828 (71.46%)	16,037 (74.95%)
3 months	9218 (57.47%)	2858 (53.35%)	12,076 (56.44%)
12 months	7552 (47.09%)	2394 (44.69%)	9946 (46.49%)

Abbreviations: *RYGB* Roux-en-Y gastric bypass, *SG* sleeve gastrectomy, *BMI* body mass index

According to current German MBS guidelines, preoperative conservative therapy, usually lasting about six months, is mandated, unless a “primary indication” (BMI > 50 kg/m^2^ or BMI > 40 kg/m^2^ in combination with type 2 diabetes) is present [4, 5]. A total of 11435 patients had a primary indication. Baseline characteristics and postoperative outcomes of the two subgroups with and without primary indication are presented in the supplementary material (Table S1).

### Seasonal Variation, First Presentation and Effects on Weight Loss

First presentation was the highest in July (*n* = 2023 (9.46%)) and the lowest in December (*n* = 1301 (6.08%)). Contrasting our hypotheses, first presentation in the first quarter of the year (January to March) was not associated with greater postoperative weight loss. The mixed model estimated a mean difference of 0.32 kg in absolute weight loss between patients presenting in the first quarter and those presenting later in the year (regression coefficient 0.32; 95%-CI: [−0.32, 0.96]; *p* = 0.3272), indicating a small and non-significant effect. Likewise, there was no evidence that surgery in the first half of the year led to better weight loss (kg) (regression coefficient − 0.22; CI: [−0.78, 0.34]; *p* = 0.4470). However, a logistic regression analysis revealed that the odds of first presentation in the first quarter of the year (January to March) were statistically significantly lower for females than for males (OR: 0.91; 95%-CI: [0.84; 0.97]; *p* = 0.0065). No evidence for an association was observed for age, baseline BMI, reported depression or presence of a primary indication for operation. Examination of the frequencies of first presentations per month showed that the months with fewest presentations were January and December, while there were two peaks with about 2,000 first presentations in February/March and in July (Table 2). The frequencies of the different surgical procedures were distributed similarly across calendar months (Table 3), indicating no relevant seasonal variation in procedure type.

**Table 2:** Frequencies of first presentations and surgeries of the entire study cohort

	First presentation, No. (%)	Surgery, No. (%)
January	1616 (7.55%)	1577 (7.37%)
February	2005 (9.37%)	1740 (8.13%)
March	1958 (9.15%)	1720 (8.04%)
April	1546 (7.23%)	1455 (6.80%)
May	1739 (8.13%)	1802 (8.42%)
June	1876 (8.77%)	2014 (9.41%)
July	2023 (9.46%)	1944 (9.09%)
August	1816 (8.49%)	1916 (8.95%)
September	1854 (8.67%)	2034 (9.51%)
October	1851 (8.65%)	2028 (9.48%)
November	1811 (8.46%)	1955 (9.14%)
December	1301 (6.08%)	1211 (5.66%)

**Table 3:** Frequencies of different operation techniques in the entire study cohort

	Proximal RYGB, No. (%)	Distal RYGB, No. (%)	SG, No. (%)
January	590 (7.06%)	7 (6.93%)	980 (7.58%)
February	662 (7.92%)	8 (7.92%)	1070 (8.27%)
March	669 (8.00%)	8 (7.92%)	1043 (8.06%)
April	595 (7.12%)	9 (8.91%)	851 (6.58%)
May	715 (8.55%)	14 (13.86%)	1073 (8.30%)
June	777 (9.29%)	4 (3.96%)	1233 (9.53%)
July	765 (9.15%)	7 (6.93%)	1172 (9.06%)
August	733 (8.77%)	6 (5.94%)	1177 (9.10%)
September	801 (9.58%)	8 (7.92%)	1225 (9.47%)
October	770 (9.21%)	15 (14.85%)	1243 (9.61%)
November	786 (9.40%)	8 (7.92%)	1161 (8.98%)
December	499 (5.97%)	7 (6.93%)	705 (4.45%)

**Fig. 2:**
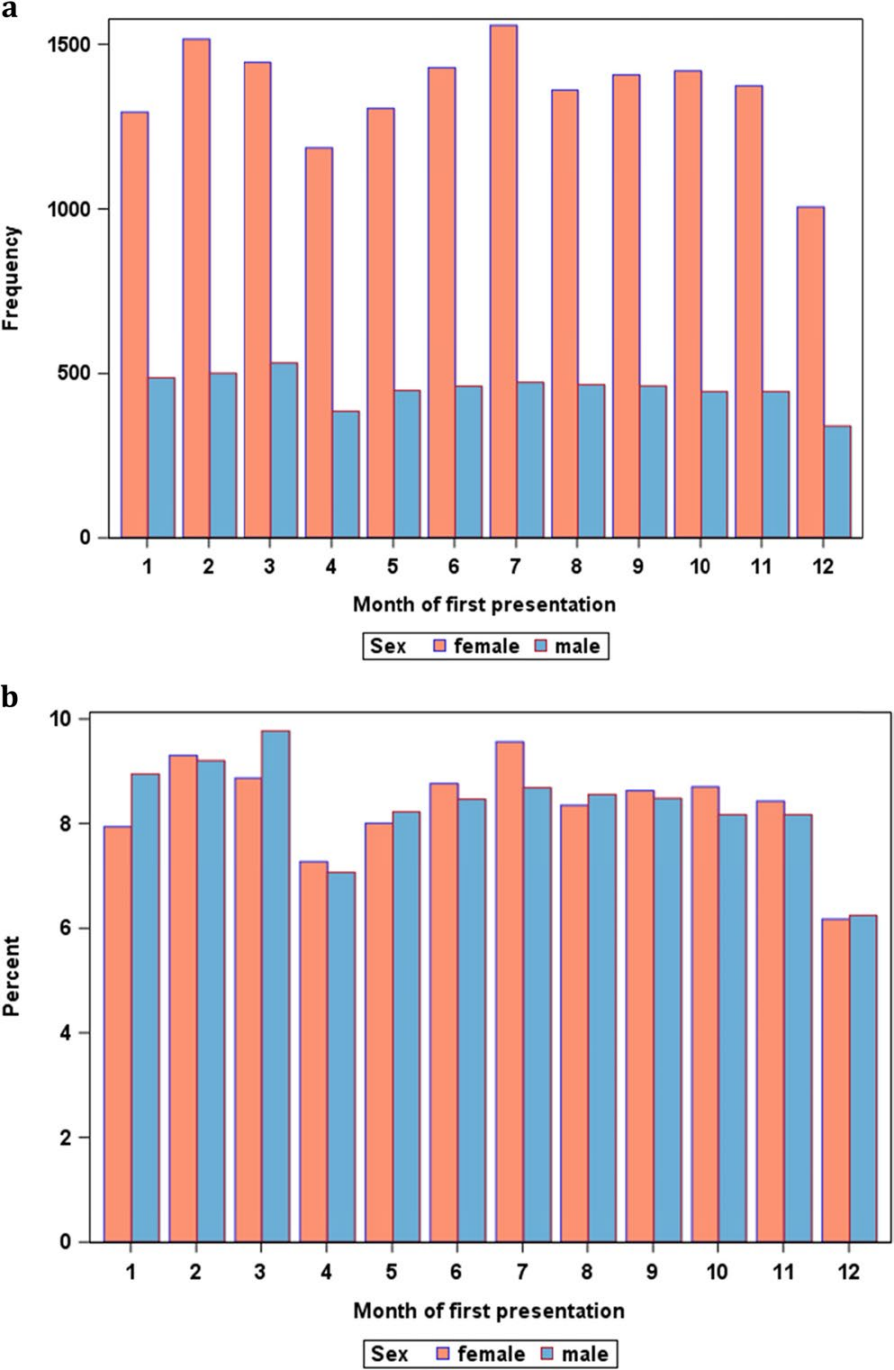
Sex-specific distributions of first presentations over the calendar year. First presentations of men and women in bariatric surgery centers are depicted in absolute numbers (**a**) and in relation to the total numbers of same sex patients presenting per year (**b**)

Because of the association of patient sex with first presentation in the first quarter of the year, the frequencies of first presentations were further examined sex-specifically (Fig. 2). Compared to the rest of the year, men first presented in MBS centers more frequently in January to March, while women’s first presentations peaked in July.

Patients with a primary indication can proceed to surgery sooner than those having to go through preoperative conservative therapy. In the StuDoQ cohort, the average time that passed between first presentation and surgery was 9.26 (7.12) months (SD) (Table 1). Patients with primary indication waited 8.69 (7.02) and those without 9.92 (7.18) months (SD) (Table S1, Supplementary Material).

**Fig. 3:**
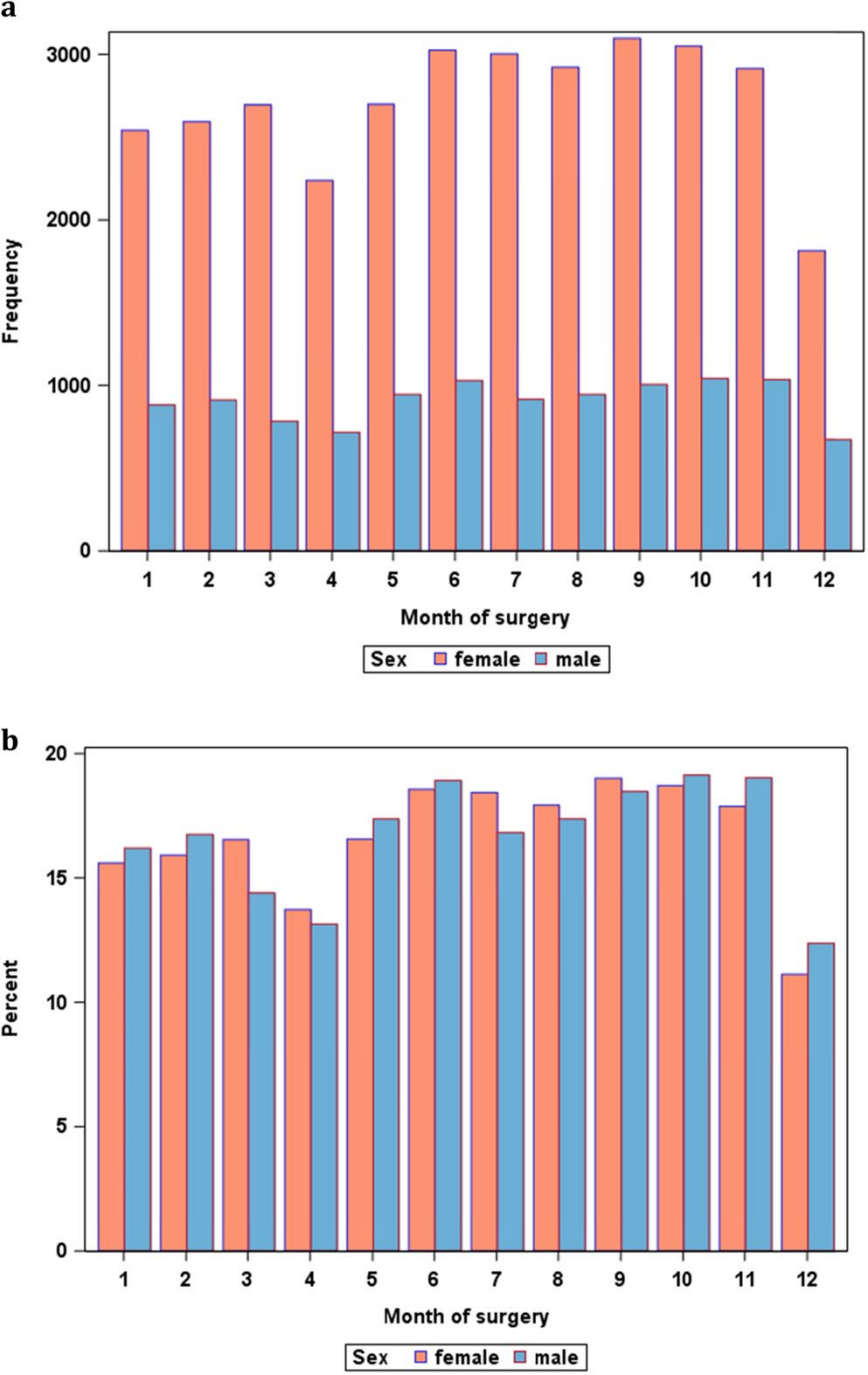
Sex-specific distributions of surgeries over the calendar year. Time of surgery for men and women in bariatric surgery centers are depicted in absolute numbers (**a**) and in relation to the total numbers of surgeries performed on same sex patients per year (**b**)

For male patients, the higher percentages of first presentation in the first quarter of the year resulted in slightly higher percentages of surgeries in October and November (Fig. 3). However, in female patients a similar trend was not seen.

## Discussion

In this large, national registry analysis of more than 21000 patients undergoing primary MBS, we found seasonal and sex-specific differences in the timing of first presentation. Women presented more frequently in spring and summer, whereas men showed a distinct peak in the first quarter of the year, likely linked to externally driven motivation. Importantly, these behavioral patterns did not translate into differences in short-term postoperative outcomes, as weight loss at 3 and 12 months remained not significantly different across all seasons. These findings suggest that while behavioral triggers influence when patients seek care, they do not affect how well they respond to surgery once treatment begins. Our study suggests women are more likely to present in spring and summer, possibly driven by increased body image awareness. Men are more often motivated by functional limitations and present more frequently at the beginning of the year, possibly linked to New Year’s resolutions. It has to be taken into account that there might be a system/appointment lag in these proposed New Year’s Resolutions. In other words, patients have the intention in January, which could lead to first presentation in an MBS clinic months later. Despite these seasonal trends in presentation, weight loss outcomes remain consistent year-round.

Sex differences in motivation for MBS are well documented. Women are frequently influenced by body dissatisfaction and sociocultural pressures related to physical appearance. Studies have shown that women undergoing MBS often report greater concern with body image and are more susceptible to societal expectations [5, 11]. Seasonal factors could also amplify these dynamics, particularly in warmer months, when revealing clothing and heightened media portrayal of ideal body types contribute to increased body image awareness [7, 9, 10]. In contrast, men tend to act in response to practical limitations, such as diminished physical function or acute health deterioration, often requiring an external trigger—including physician advice or calendar-based motivation (e.g., New Year’s resolutions) [1, 2, 9].

Data from the German StuDoQ registry aligns with these conceptual frameworks: male patients exhibit a presentation peak in the first quarter of the year, whereas female presentations are more common in summer. This distinction suggests differing underlying motivators—goal-oriented, event-driven planning in men versus body-centric, psychosocial triggers in women.

Despite these divergent presentation patterns, no evidence has been found for corresponding seasonal variation in postoperative outcomes. Weight loss at 3 and 12 months remains consistent across all seasons. Importantly, given the average delay of approximately nine months between first presentation and surgery in this cohort, the season of presentation and the season of surgery are frequently decoupled. Therefore, these variables were analyzed separately in the statistical models. This approach allowed us to distinguish seasonal patterns in patient consultation behavior from potential seasonal influences related to the timing of surgery itself. These findings challenge the assumption that early-year motivation or summertime treatment inherently enhances outcomes, underscoring the robustness of bariatric protocols across temporal contexts. Additionally, the confidence interval around the estimated difference in weight loss between seasonal groups was relatively narrow, suggesting that any true effect is small and unlikely to represent a clinically meaningful difference in postoperative weight loss. Given the large sample size of this study, it is also unlikely that the absence of a significant association is due to insufficient statistical power. These findings contrast the studies done by Cambras et al. [9, 10] that there might be a seasonal difference in weight loss after MBS.

Our study did not reveal seasonal clustering of complications or suboptimal outcomes. In contrast, Scandinavian data from SOReg reported increased rates of postoperative complications following the summer holiday period [13]. These findings may reflect differences in system-level continuity of care and highlight the importance of structured follow-up programs that mitigate the effects of staff turnover or seasonal resource fluctuations.

Our results could be helpful in daily practice and counseling of obesity medicine specialists and bariatric surgeons. Some limitations and potential confounders need to be addressed. Since women mostly pursue bariatric and metabolic surgery it is no surprise that in our dataset approximately 75% of the included patients are women. This could lead to potential confounding bias. Secondly aspects like the observational design of this study, registry constraints, absence of motivational data and missing data could be confounding factors that could potentially hamper the interpretation and generalizability of the study results. In addition, center-level effects could not be examined because the registry extract available for this study did not contain center identifiers, preventing adjustment for potential variation in practice patterns between participating centers. Finally, it should be considered that care pathways and seasonal behaviors may differ among regions and could also be different in other countries.

It is important to recognize the temporal and sex-specific drivers of presentation, which can lead to the development of personalized counseling strategies. For example, clinicians may address aesthetic concerns and body image more proactively with female patients during spring and summer. Secondly, structured campaigns could leverage seasonal trends—such as New Year’s resolutions for men and pre-summer preparation for women—to increase patient engagement. Finally, bariatric education and preparation programs should include modules that address psychological readiness, seasonally linked stressors, and gender-sensitive motivation.

## Conclusions

Seasonal and sex-specific behavioral patterns could influence the timing of bariatric consultation, but there is no evidence for a difference in postoperative surgical outcomes. These insights underscore the importance of individualized patient engagement strategies. By recognizing and addressing the nuanced motivations behind treatment-seeking behavior, healthcare providers can better align support systems with patient needs, enhancing the efficacy and inclusivity of bariatric care.

## Supplementary Information

Below is the link to the electronic supplementary material.

Supplementary Material 1 (PDF 83.0 KB)

## Data Availability

No datasets were generated or analysed during the current study.
