# Supplementary material for: New Year’s Resolution or Spring Feelings? Effect of Month and Season on First Presentation and Short-Term Outcome in Metabolic and Bariatric Surgery
Source: Obes Surg. 2026 Apr 16;36(5):2527–33. doi: 10.1007/s11695-026-08665-7 (PMC13222258; doi:10.1007/s11695-026-08665-7)
Supplement: Supplementary file 1 — Supplementary Material 1 (PDF 83.0 KB) [file 11695_2026_8665_MOESM1_ESM.pdf]

Table S1: Baseline characteristics and postoperative outcomes of the study cohort with and without primary indication for metabolic-bariatric surgery

Variable	Primary indication (n=11435)	No primary indication (n=9961)
Age at surgery, mean (SD), years	43.94 (11.67)	43.48 (11.18)
Female Sex, No. (%)	8050 (70.40%)	7989 (80.22%)
Proximal RYGB, No. (%)	4026 (35.21%)	4336 (43.53%)
Distal RYGB, No. (%)	46 (0.40%)	55 (0.55%)
SG, No. (%)	7363 (64.39%)	5570 (55.92%)
First presentation in Jan-Mar, No (%)	2931 (25.63%)	2648 (26.58%)
Delay between first presentation and surgery, mean (SD), months	8.69 (7.02)	9.92 (7.18)
BMI, mean (SD), kg/m^2		
Baseline	53.57 (7.05)	43.76 (3.69)
3 months	43.06 (6.59)	35.34 (4.15)
12 months	36.08 (6.44)	29.51 (4.28)
Body weight, mean (SD), kg		
Baseline	141.26 (27.11)	141.16 (26.91)
3 months	113.71 (23.46)	113.68 (23.14)
12 months	95.31 (21.73)	95.11 (21.61)
Depression, No. (%)		
Baseline	3300 (28.86%)	2817 (28.28%)
3 months	2543 (22.24%)	2100 (21.08%)
12 months	2442 (21.36%)	1979 (19.87%)
Type 1 Diabetes, No. (%)		
Baseline	136 (1.19%)	18 (0.18%)
3 months	132 (1.15%)	31 (0.31%)
12 months	115 (1.01%)	35 (0.35%)
Type 2 Diabetes, No. (%)		
Baseline	4700 (41.10%)	442 (4.44%)
3 months	3826 (33.46%)	575 (5.77%)
12 months	3079 (26.93%)	470 (4.72%)
Joint pain, No. (%)		
Baseline	8635 (75.51%)	7402 (74.31%)
3 months	6757 (59.09%)	5319 (53.40%)

12 months	5670 (49.58%)	4276 (42.93%)

Abbreviations: RYGB = Roux-en-Y gastric bypass; SG = sleeve gastrectomy; BMI = body mass index.
